# Cold water immersion of the hand and forearm during half-time improves intermittent exercise performance in the heat

**DOI:** 10.3389/fphys.2023.1143447

**Published:** 2023-06-08

**Authors:** Manami Iwahashi, Yudai Chaen, Takuma Yanaoka, Yasutsugu Kurokawa, Hiroshi Hasegawa

**Affiliations:** Graduate School of Humanities and Social Sciences, Hiroshima University, Hiroshima, Japan

**Keywords:** hot environment, body cooling, intermittent exercise performance, repeated sprint performance, core temperature, thermal sensation

## Abstract

The present study aimed to investigate the effect of cold water immersion of the hand and forearm during half-time (HT) on intermittent exercise performance and thermoregulation by imitating intermittent athletic games in the heat. In a randomized crossover design, 11 physically active men performed the first half (first and second block) and second half (third and fourth block) intermittent cycling exercise protocol, which consisted of a 5-s maximal power pedalling (body weight × 0.075 kp) every minute separated by 25-s of unloaded pedalling and rest (30 s) in the heat (33°C, 50% relative humidity). The two-halves were separated by a 15-min HT. During HT, the participants were assigned to the CON (sedentary resting) or COOL (immersion of hands and forearms in cold water at 15–17°C) condition. The mean power output in the second half was significantly greater (third and fourth block: *p* < 0.05) in the COOL than in the CON condition. Moreover, there was a significant decrease in the rectal (0.54 ± 0.17°C, *p* < 0.001) and mean skin (1.86 ± 0.34°C, *p* < 0.05) temperatures of the COOL condition during HT. Furthermore, the heart rate (16 ± 7 bpm, *p* < 0.05) and skin blood flow (40.2 ± 10.5%, *p* < 0.001) decreased at the end of HT in the COOL condition. In the second half, thermal sensation was more comfortable in the COOL condition (*p* < 0.001). Cold water immersion of the hand and forearm during HT improved physiological and reduced perceived heat stress. Moreover, it prevented a reduction in intermittent exercise performance in the second half.

## 1 Introduction

In many team sports, athletes frequently perform in the heat and are required to sustain exercise performance. Compared to temperate environments, the core (T_c_) and mean skin (T_sk_) temperatures increase in the heat faster, resulting in increased cardiovascular, metabolic, and thermal perceptual loads and decreased endurance and intermittent exercise performance (Nybo et al, 2014; Drust et al, 2005). Therefore, it is necessary to actively apply cooling strategies that can reduce both the actual and perceived heat stress to achieve high exercise performance even in the heat.

Several intermittent team sports have short periods of rest between exercises, such as half-time (HT), and intervals during which cooling strategies can be employed. Furthermore, the HT may be an important time for cooling interventions because repeated sprint performance noticeably decreases in the second half (Mohr et al, 2010). However, few studies have examined the effects of body cooling on high-intensity exercise performance and physiological and perceptual responses with a focus on short periods of rests between exercises. Previous studies reported that whole-body cold-water immersion, which appears to be the most effective body cooling strategy, lowered T_c_ during simulated HT, and the decrease persisted until the end of the subsequent 30-min endurance exercise (Peiffer et al, 2010) or 40-min intermittent exercise (Egaña et al, 2019), resulting in the suppression of declines in high-intensity exercise performance. However, whole-body cold water immersion requires a large amount of ice and a bathtub and is impractical in athletic settings. Another study demonstrated that ice slurry ingestion, which is a more frequent cooling strategy in actual sport competitions, during simulated HT successfully lowered T_c_ (Onitsuka et al, 2015). However, the reduction in T_c_ after ice slurry ingestion was brief and converged within 10 min during subsequent intermittent exercise; moreover, no improvements in intermittent exercise performance was observed (Onitsuka et al, 2015). This trend has also been reported in several studies (Gerrett et al, 2016; Thomas et al, 2019), suggesting a trivial effect of reductions in only T_c_ on intermittent exercise performance. Aldous et al (2018) found that mixed-method cooling (ice slurry and ice packs placed on the quadriceps and hamstrings) reduced T_c_, T_sk_, and thermal sensation (TS), which were maintained throughout the subsequent 45 min of intermittent exercise. Chaen et al (2019) reported that wearing a cooling vest during simulated HT reduced T_sk_ and TS, and the decreased TS was maintained throughout the subsequent 30-min of intermittent exercise. In addition, both studies reported improvements in intermittent exercise performance (Aldous et al, 2018; Chaen et al, 2019). These studies explain the importance of maintaining a low thermophysiological responses during intermittent exercises to attenuate heat-induced reductions in intermittent exercise performance. In addition, sports competitions require cooling interventions that are simple, short-lived, and have a high cooling capacity.

Cold water immersion of the hand and forearm is an effective cooling method for lowering T_c_ and may improve exercise performance. Several studies have reported that 10–15 min of forearm water immersion significantly reduces core and mean skin temperatures after exercise in the heat (Khomenok et al, 2008; Barr et al, 2011). A possible mechanism underlying this reduction in temperature may be the large potential area for heat transfer, as a high surface area-to-mass ratio and arteriovenous anastomoses (AVA) in the hands, together with superficial veins up to the elbow, form a specialized heat exchange organ that allows for large variations in local blood flow (Vanggaard et al, 2012). Consequently, the cooled blood returns to the core through the superficial veins, which helps reduce T_c_ (Livingstone et al, 1989). In a recent study, cold water immersion of the hand and forearm for 15 min in exercise-induced hyperthermia prolonged the time to exhaustion in subsequent exercise compared with the no-cooling condition by decreasing T_c_ and T_sk_ (Nakamura et al, 2020). Although this previous study examined the effects of brief cold water immersion of the hand and forearm, the exercise protocol employed was not similar to actual exercise performance. Thus, it is necessary to investigate the effects of cold water immersion of the hand and forearm in an exercise protocol that closely resembles the actual competition, which may include repeated sprints.

Cooling of the palmar region, including the AVA and forearm, which have an advantage in heat dissipation, may contribute to the return of a large amount of cool blood to the core region, alleviating heat stress or improving exercise performance. Therefore, the present study focused on the HT cooling strategy and aimed to investigate the effect of cold water immersion of the hand and forearm during HT on intermittent exercise performance by imitating intermittent athletic games. We hypothesized that cold water immersion of the hand and forearm during HT would improve subsequent intermittent exercise performance by decreasing T_c_ and T_sk_ and reducing perceived heat stress.

## 2 Methods

### 2.1 Participants

Eleven healthy male soccer players (age: 21 ± 1.6 years, height: 169.6 ± 9 cm, body mass [BM]: 61.7 ± 4.5 kg) participated in this study, and they train 6 days a week. The sample size was estimated using G*Power 3 (Faul et al, 2007), using mean power output data (Cohen’s d: 1.89) from a previous study (Chaen et al, 2019). The required sample size was calculated using an alpha and power level of 0.05 and 0.8. Based on these results, a minimum of nine participants was required to detect an improvements in exercise performance. They abstained from alcohol and caffeine consumption 24 h before the experiment. All trials took place during the winter season (mean ambient temperature: 7°C) to avoid the influence of heat acclimatization. This study was approved by the Ethics in Human Research Committee of Hiroshima University (Approval number: 02-21), and all participants signed an informed consent before the start of the study.

### 2.2 Experimental design

After familiarization, participants completed two experimental trials in a randomized and crossover design. Familiarization and each experimental trial were separated by at least 7 days and completed at the same time of day for each participant to avoid any circadian rhythm-related variations. Participants were asked to avoid altering their regular lifestyle habits, exercises, and diet throughout the study.

In the experimental trials, participants performed two 30-min halves separated by a 15-min HT. During HT, participants either maintained sedentary rest (CON) or immersed their hands and forearms in cold water (15–17°C) up to elbow (COOL). In the familiarization trial, they performed the same protocol as the CON. Familiarization and experimental trials took place in a climate environmental chamber in the heat (33°C, 50% relative humidity).

### 2.3 Exercise protocol

**FIGURE 1:**
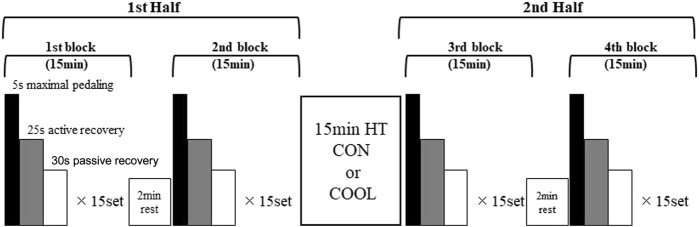
Exercise protocol, HT: half-time.

All sessions were completed with the use of a cycling ergometer (POWERMAX-V3 PRO; Konami Sports Life, Japan). Participants completed warm-up exercises consisting of a 7-min cycling (BM × 0.01 kp, 80 rpm) and 3 sets of 5-s maximal pedalling and 55-s recovery as described below at 2-min before the exercise protocol. Participants completed a laboratory-based intermittent exercise protocol designed to replicate the demands of actual intermittent athletic games. The protocol consisted of two 30-min halves separated by a 15-min HT. A 30-min half was comprised of two 15-min blocks separated by a 2-min rest period. A block consisted of a 5-s maximal pedalling (BM × 0.075 kp), 25-s active recovery (no load, 80 rpm), and 30-s passive recovery with the cycles repeated over 15-min (Figure 1). During all sessions, participants were allowed to consume water (33°C) up to 750 mL during the 2-min rest and HT, and all participants drank all the water. The temperature of the drinking water was set to the room temperature to eliminate any effect on Tc.

### 2.4 Cooling intervention

During HT, participants were assigned to either CON (maintain sedentary rest) or COOL (immerse their hands and forearms) condition. Participants undertook both interventions in the heat (33°C, 50% relative humidity). In the COOL condition, participants sat on the floor in chamber and immersed their hands and forearms up to the elbows in cold water using two buckets. To maintain a water temperature at 15–17°C (Barwood et al, 2009), monitoring was carried out using digital thermometer (TT-508, TANITA, Japan), and ice cubes were added as necessary. Given 1-min was required to get on and off the ergometer, the actual cooling time was 14-min.

### 2.5 Measurements

#### 2.5.1 Performance index

The mean and maximal power outputs during 5-s maximal pedalling were measured as indices of the intermittent exercise performance. The mean power output and peak rpm during 5-s maximal pedalling were measured using a built-in computer in the cycling ergometer (POWERMAX-V3 PRO; Konami Sports Life, Japan) (Chaen et al, 2019). The maximal power output during 5-s maximal power pedalling was calculated by the following equation. Maximal power output=BM×0.075Kp×peak rpm

#### 2.5.2 Physiological index

Rectal temperature (T_re_), T_sk_, heart rate (HR), skin blood flow (SkBF), urine specific gravity, and nude BM were measured in this study. T_re_ was recorded using a thermistor (LT-ST08-21, Nikkiso-Therm, Japan) from a depth of 10–12 cm past the anal sphincter at 1-min intervals. T_sk_ were measured by attaching a thermistor probe (LT-ST08-12, Nikkiso-Therm, Japan) on the chest, upper arm, and thigh. The T_sk_ was calculated using the formula developed by Roberts et al (1977) and evaluated by Taylor et al (2014): $T_{sk}=\left(0.43\times chest\right)+\left(0.25\times upper\;arm\right)+\left(0.32\times thigh\right).$

Participants also wore a HR monitor (V800, Polar Electro, Finland) that was attached before entering the environmental chamber. SkBF was measured with a laser doppler flowmeter (ALF21, ADVANCE, Japan) placed on upper arm after entering the environmental chamber, and measurements were taken only from the end of first half of exercise to the end of HT. The SkBF values were normalized by using the value obtained from the end of the first half. Tre, Tsk, HR, SkBF were measured every 3 min during the experiment. The urine specific gravity was measured using a digital urine specific gravity refractometer (UG-D, Atago Co., Ltd., Japan) before the experiment. Nude BM was measured using a scale (UC-300, A&D Co., Ltd., Japan) before the experiment and used to estimate dehydration rate. The sweat rate and dehydration rate were calculated using the following formula: Sweat rate (kg) = (nude body mass before the trial [kg] + fluid ingestion [kg]—nude body mass after the trial [kg]). Dehydration rate (%) = body sweat rate [kg]/nude body mass before the trial [kg] × 100.

#### 2.5.3 Perceptual index

TS and thermal comfort (TC) were measured every 3 min during the experiment. TS and TC were rated using a 13-point scale that ranged from −6 (very cold) to 6 (very hot) and −6 (very uncomfortable) to 6 (very comfortable), respectively (Olesen and Brager, 2004). Rating of perceived exertion (RPE, 6 [no exertion] to 20 [maximal exertion]) (Borg, 1982) was measured every 3 min.

### 2.6 Statistical analyses

All statistical calculations were performed using SPSS version 25.0. The accepted level of significance for all analyses was *p* < 0.05. All data were presented as mean ± SD. The Shapiro-Wilk test was used to check for normality of distribution. Mean power output, maximal power output, T_re_, T_sk_, HR, and SkBF were analyzed using two-factor analysis of variance (conditions × time). Where significant main effects or interactions were found, values were subsequently analyzed using Bonferroni multiple comparisons test. TS, TC, and RPE were analyzed using Friedman analysis of variance and Wilcoxon’s matched pairs tests. Dehydration rate and urine specific gravity were analyzed using a paired *t*-test. All variables were also analyzed using Cohen’s d effect sizes, whereby ≥0.8 was categorized as a large effect, 0.5 to 0.79 as a moderate effect, and <0.49 as a small effect (Cohen, 1988).

## 3 Results

### 3.1 Performance

**FIGURE 2:**
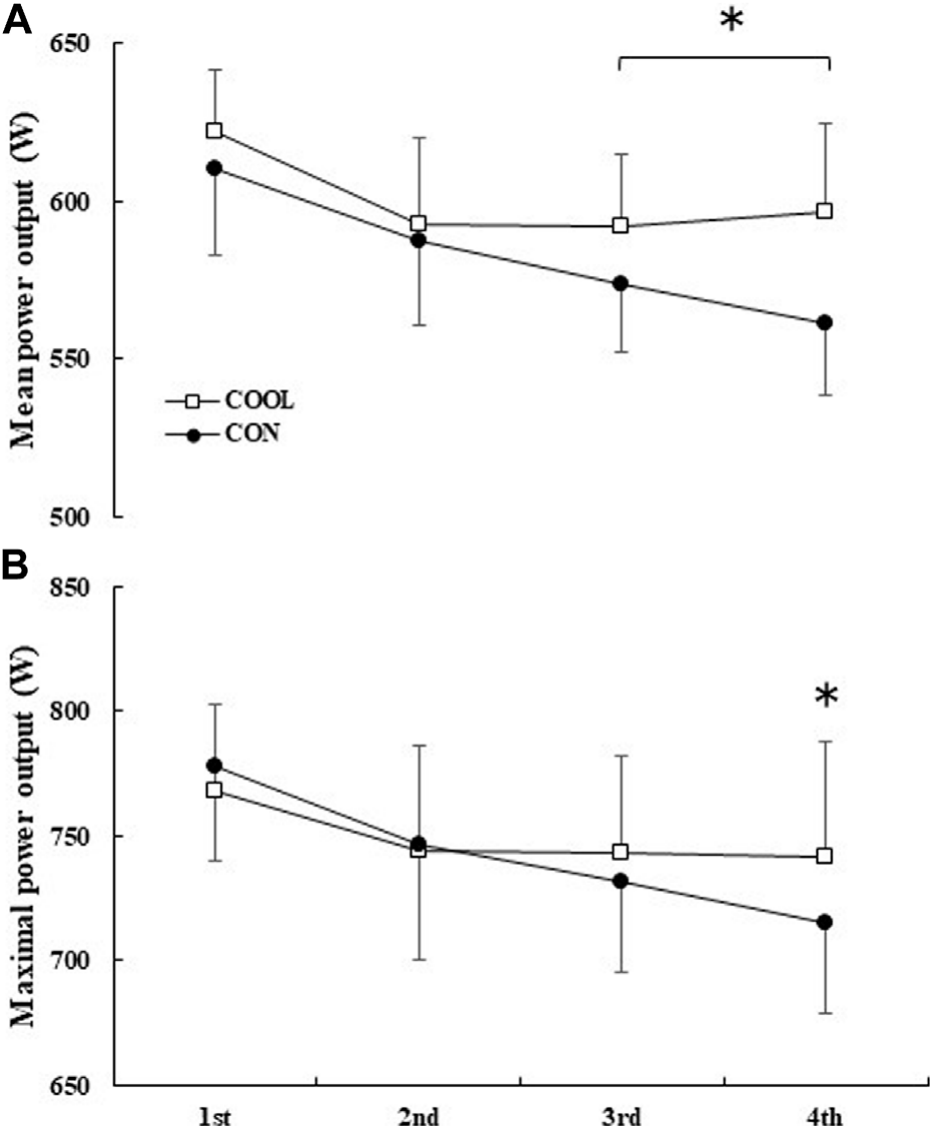
The mean **(A)** and maximal **(B)** power output during exercise. The values are shown as mean ± SD (n = 11). * Significant difference between the conditions (*p* < 0.05).

The within-subject coefficients of variation for the mean power output during the first half was 1.02 ± 1.2%. The mean power output and maximal power output showed a condition × time interaction (both *p* = 0.005) (Figure 2). The mean power output during the third and fourth blocks was significantly decreased in both CON (third block: *p* = 0.019, d = 1.46; fourth block: *p* = 0.001, d = 1.95) and COOL (third block: *p* = 0.003, d = 1.41; fourth block: *p* = 0.037, d = 1.06) conditions compared to that during the first block. However, the mean power output in the third and fourth blocks was significantly greater (third block: *p* = 0.020, d = 0.8; fourth block: *p* = 0.003, d = 1.38; Figure 2) in the COOL condition (third block: 592 ± 22 W, fourth block: 596 ± 27 W) than in the CON condition (third block: 574 ± 22W, fourth block: 561 ± 22W). The maximal power output in the third and fourth blocks was significantly decreased compared to that in the first block in the CON condition (third block: *p* < 0.001, d = 1.27; fourth block: *p* < 0.001, d = 1.91) but not in the COOL condition (third block: *p* = 0.074, d = 0.68; fourth block: *p* = 0.285, d = 0.64). Furthermore, the maximum power output in the fourth block was significantly greater (*p* = 0.049, d = 0.71) in the COOL condition (741 ± 46 W) than in the CON condition (715 ± 27 W).

### 3.2 Body temperature

**FIGURE 3:**
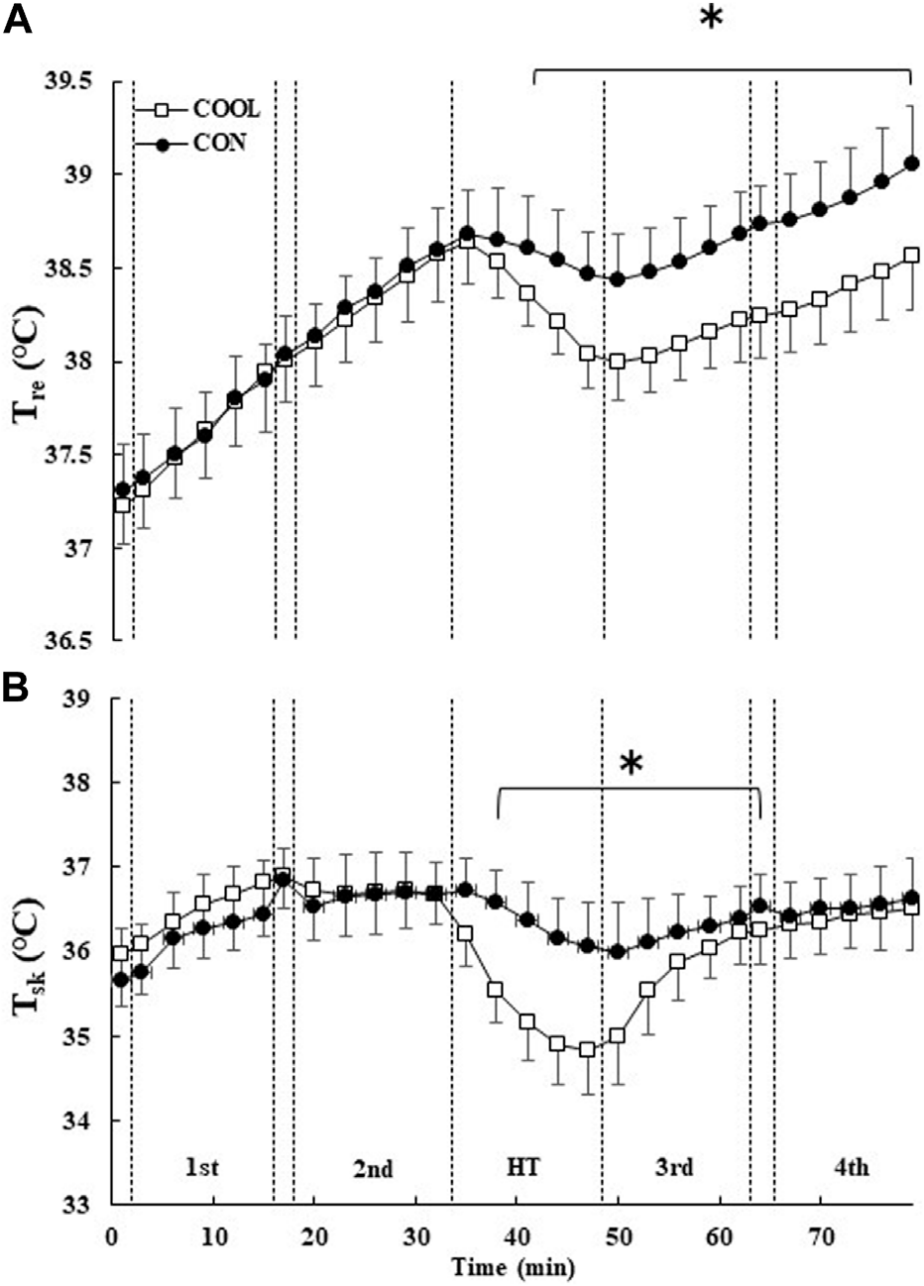
The rectal (T_re_; **(A)**) and mean skin (T_sk_; **(B)**) temperatures throughout the experimental protocol. The values are shown as mean ± SD (n = 11). * Significant difference between the conditions (*p* < 0.05).

T_re_ and T_sk_ showed a condition × time interaction (both *p* < 0.001) (Figure 3). No significant differences in T_re_ and T_sk_ were observed between the conditions before and after the first half. T_re_ significantly decreased by 0.54 ± 0.17°C during HT in the COOL condition compared to that in the CON condition (*p* < 0.001, d = 2.15). In addition, T_re_ was significantly lower in the COOL condition than in the CON condition in the third and fourth blocks, and the decrease was sustained until the end of exercise (*p* < 0.05; Figure 3). T_sk_ was significantly lower in the COOL condition than in the CON condition during HT and for most of the third block (*p* < 0.05; Figure 3).

### 3.3 Heart rate and skin blood flow

**FIGURE 4:**
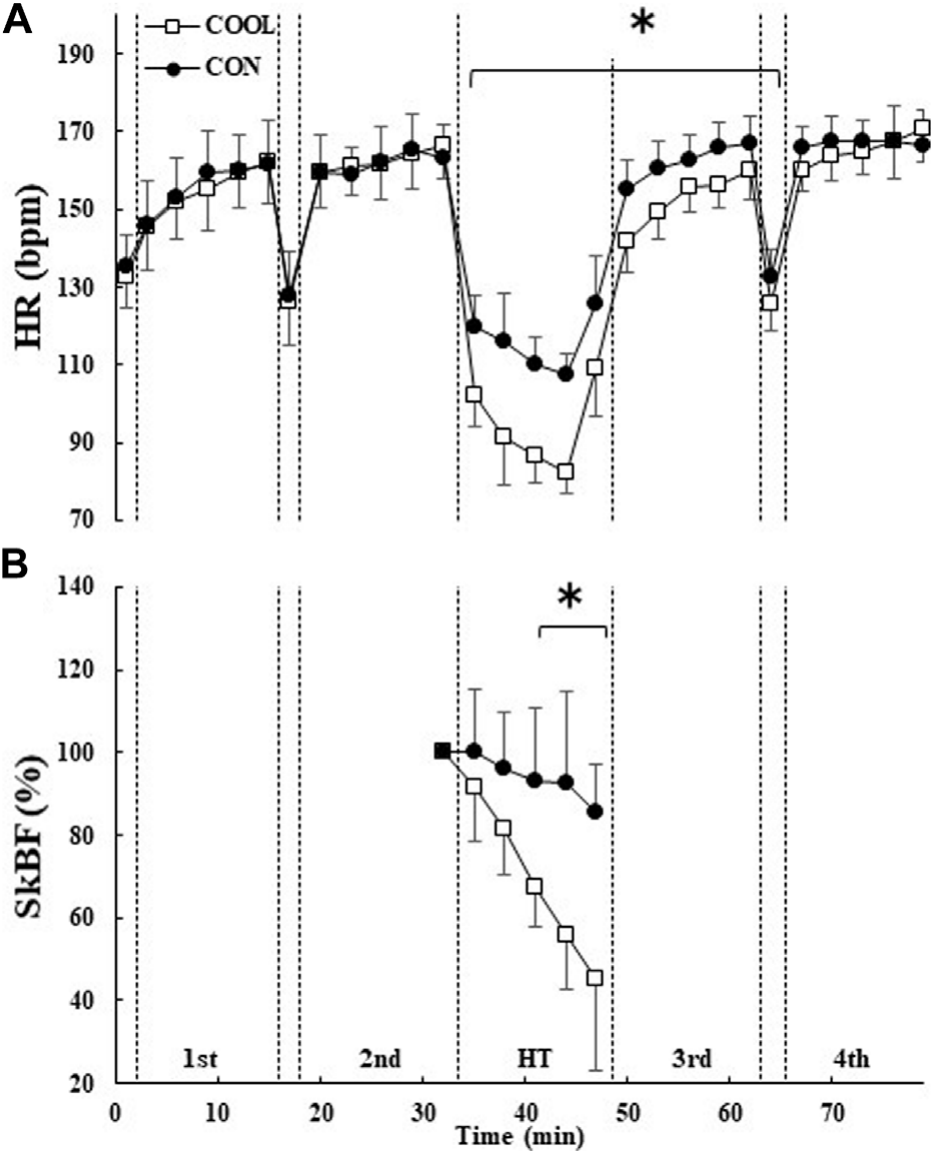
The heart rate (HR; **(A)**) throughout the experimental protocol and forearm skin blood flow (SkBF; **(B)**) during half-time. The values are shown as mean ± SD (n = 11). * Significant difference between the conditions (*p* < 0.05).

HR and SkBF showed a condition × time interaction (both *p* < 0.001) (Figure 4). No significant difference in HR was observed between the conditions before and after the first half. HR was significantly lower in the COOL condition than in the CON condition during HT and in the third block (*p* < 0.05; Figure 4). SkBF was also significantly lower in the COOL condition than in the CON condition during HT (*p* < 0.05; Figure 4).

### 3.4 Body fluid balance

Prior to the experiment, the participants were adequately hydrated. The urine specific gravity (CON: 1.018 ± 0.008, COOL: 1.018 ± 0.003) and dehydration rate (CON: 2.54 ± 0.94%, COOL: 2.28 ± 0.84%) did not differ between the conditions.

### 3.5 Perceptual index

**FIGURE 5:**
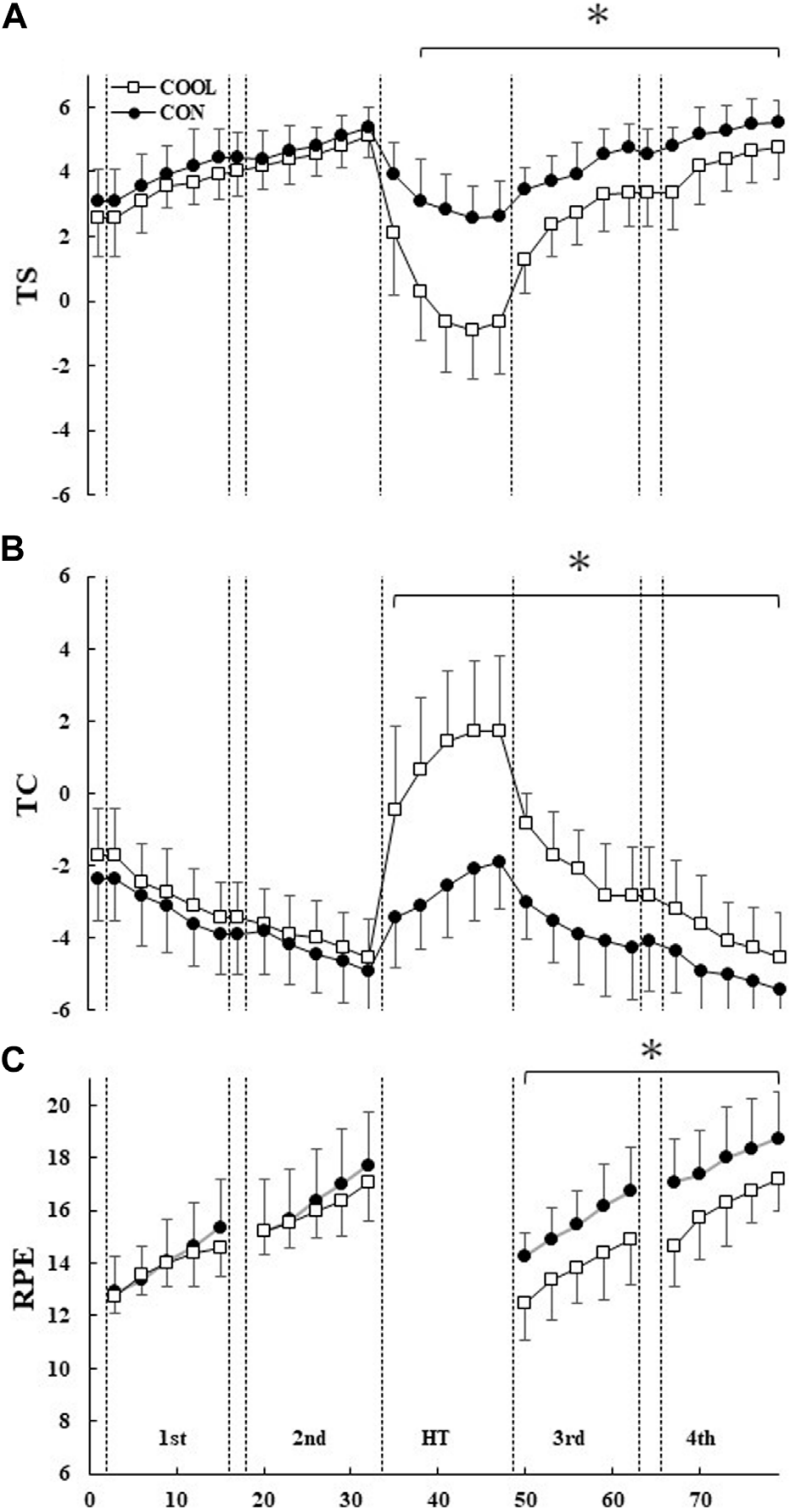
The thermal sensation (TS; **(A)**), thermal comfort (TC; **(B)**), and rating of perceived exertion (RPE; **(C)**) throughout the experimental protocol. The values are shown as mean ± SD (n = 11). * Significant difference between the conditions (*p* < 0.05).

No significant differences in TS, TC, and RPE were observed between the conditions before and after the first half (Figure 5). TS was significantly lower in the COOL condition from HT to the fourth block (*p* < 0.05). On the contrary, TC was significantly higher in the COOL condition from HT to the fourth block (*p* < 0.05). During the second half, RPE was significantly lower in the COOL condition than in the CON condition (*p* < 0.05).

## 4 Discussion

In this study, we investigated the effect of cold water immersion of the hand and forearm during HT on intermittent exercise performance by imitating intermittent athletic games. The main results of our study showed that cold water immersion of the hand and forearm during HT significantly decreased T_re_ and T_sk_. Moreover, it reduced thermal and cardiovascular strain and subjective sensation, resulting in improved intermittent exercise performance in the second half. It has been reported that high-intensity exercise performance in the second half of intermittent team sports is decreased in the heat (Mohr et al, 2010). Therefore, the improvement of intermittent exercise performance in the second half using the practical cooling method of cold water immersion of the hand and forearm during HT is a very important finding for sports competitions in a hot environment.

Interestingly, the limited time and simple cooling method of cold water (15–17°C) immersion of the hand and forearm during HT lowered the T_re_ and sustained it until the end of the second half of the exercise. Since excessive elevation of T_c_ is a major factor in impaired exercise performance in hot condition, it is essential to suppress the increase in T_c_ in athletic settings. Cold water immersion of the hand and forearm has been shown to be effective in lowering T_c_ because the palmar AVA function allows cooled blood to circulate through the body. The results of this study were consistent with previous studies that successfully lowered T_re_. Furthermore, the reduction in T_re_ by 0.54°C in our study was significantly greater than in other studies which combined methods of hand cooling (9°C) and ice slurry ingestion during a half-time interval, which reduced T_re_ by 0.2°C (Soo et al, 2019), or cold water immersion (10°C) of the hand and forearm for 15 min, which reduced T_re_ by 0.45–0.48°C (Khomenok et al, 2008), or water immersion (19°C) of the hand and forearm with ice vest for 15 min, which reduced T_re_ by 0.2°C (Barr et al, 2011). These differences in reduction of T_c_ may be due to a combination of the degree of increase in body temperature during exercise, environmental temperature and humidity, cooling site, cooling methods, cooling time, and changes in blood flow. Therefore, cold water immersion of the hands and forearms used in this study was shown to be an effective method of removing heat stress after exercise in a hot environment during HT and may improve intermittent sprint performance in the second half of exercise by moderately lowering T_c_. Moreover, it has been reported that when T_re_ is lowered without a decrease in T_sk_ using body cooling pre or per exercise (Onitsuka et al, 2015; Bongers et al, 2017), the increase in T_re_ is enhanced during subsequent exercise. This is because when only T_re_ is lowered, the core-to-skin temperature gradient is lowered, and the ability to transfer heat to the environment is decreased (Siegel et al, 2012). The cold water immersion of the hand and forearm used in this study was able to simultaneously lower not only T_re_ but also T_sk_, thus suppressing the increase in T_re_ until the end of the second half of the exercise and contributing to high exercise performance.

One of the factors contributing to decreased exercise performance in the heat is an increase in peripheral SkBF for heat dissipation and a consequent decrease in blood supply to the skeletal muscles (Bell et al, 1983). To alleviate these cardiovascular loads, it is important to improve the elevated skin temperature during rest. In the present study, T_sk_ was reduced by cold water immersion of the hands and forearms during HT. Reduced Tsk has been reported to potentially relieve the cardiovascular load, increase blood supply to skeletal muscles (Sleivert et al, 2001; Gonzalez-Alonso & Calberts. 2003), and promote the recovery of central blood volume (Wilson et al, 2002). In the present study, HR dropped significantly during HT, and this lowered HR was sustained until the end of the third block of exercise, suggesting that cold water immersion of the hand and forearm reduced the cardiovascular load and improved oxygen and energy delivery to the skeletal muscle, resulting in increased power output in the second half. However, because we did not measure direct indices such as skeletal muscle oxygen dynamics or muscle blood flow, we could not clarify whether the improved oxygen and energy supply to the skeletal muscle resulted in a higher power output. In addition, high-power exertion in the second half of the exercise is thought to increase the cardiovascular load. Although the significant decrease in HR disappeared in the middle of the second half of the exercise, it should be noted that the HR was not higher than that in the CON condition until the end of the exercise, indicating that the high cardiovascular load may have been offset by the cooling effect.

It is also important to improve the perceived heat stress during exercise. In particular, the decline in self-paced exercise performance in the heat has been shown to be potentially due to the hypothalamus sensing elevated heat perception and reducing exercise intensity to avoid exceeding the critical body temperature (Tucker & Noakes. 2009). This suggests that thermal perception plays a major role in determining the motivation to continue exercising in the heat. In the present study, significant improvements in both TS and TC were observed in the COOL condition compared with those in the CON condition from the HT to the end of the fourth block. An increase in T_sk_ during exercise elevates thermal perception and thermal discomfort. Therefore, improvement in thermal perception is thought to be influenced by the improvement in T_sk_ (Flouris & Schlader. 2015). T_sk_ in the present study decreased by approximately 2°C in the COOL condition and was significantly lower than that in the CON condition. A previous study in which cooling vests were worn during HT with a protocol similar to that of the present study reported a reduction in T_sk_ to the same level as in the present study as well as improved intermittent exercise performance due to lowered thermal perception (Chaen et al, 2019). This is based on the mechanism that alleviates perceived heat stress, reduces selective reduction in exercise intensity, and improves exercise performance in the heat (Faulkner et al, 2015; Schumit et al, 2017). Therefore, the present study also caused a decrease in T_sk_ as well as decrease in thermal perception, which allowed for a higher power output while maintaining a lower RPE.

Several studies have evaluated different cooling methods during intermittent exercise to reduce the thermal load and improve exercise performance (Duffield et al, 2013; Holm et al, 2015; Aldous et al, 2018; Chalmers et al, 2019). These studies were well designed, used athletes as study participants, and assessed performance in heat and actual game-style exercises. Cold water immersion during short breaks appears to be the most effective cooling method for reducing thermoregulatory strain and improving exercise performance (Egaña et al, 2019; Peiffer et al, 2010). However, it is difficult to apply this method during HT because of the requirement of a large amount of ice and bathtubs. Moreover, cooling the lower extremities, including the thighs, is not suitable for intermittent team sports with HT because it cools the main active muscles, which increases the risk of poor athletic performance immediately after the start of the second half (Yanaoka et al, 2021) and sports injuries (Rahnama et al, 2002). Wearing cooling garments and/or ingesting cold beverages is more practical; however, employing them during short breaks is less effective than cold water immersion. Previous studies have investigated the efficacy of isolated (cooling vest (Chaen et al, 2019) and ice slurry ingestion (Onitsuka et al, 2015)), as well as those of mixed-methods (placing ice packs on the thigh and ice slurry ingestion (Aldous et al, 2018) and hand cooling, ice vest, and ice slurry ingestion (Soo et al, 2019)) cooling strategy during 15-min breaks; however, these studies reported no significant reductions in T_re_ and/or improvements in exercise performance. The magnitude of cooling-induced alterations in physiological and performance variables depends on the cooling power (Minett et al, 2011; Tyler et al, 2015). Therefore, more aggressive and feasible cooling strategies that can be used during short breaks are required. We successfully implemented an effective strategy using hand and forearm cooling over a limited and short period.

Although various countermeasures (such as cold water immersion, cooling vest, ingestion of liquid/ice slurry, cooling with a large fan, and using cold wet towels) prevent the increase in T_c_ and decline exercise performance, few studies have successfully observed both a decrease in T_c_ and an increase in exercise performance in a hot environment. Compared to the effectiveness of previously analyzed cooling interventions on the physiological responses and performance of athletes during intermittent exercise in the heat, the cooling strategy that involves using hand and forearm cooling, which we used, has sufficient cooling power and practicality. The degree of reduction in T_c_ in the second half was greater with a short cooling period at HT, and the reduction in exercise performance associated with the increase in T_c_ during the second half of the heat was suppressed. Moreover, in intermittent team sports, such as soccer and rugby, the resting time between exercises is short. Thus, convenient interventions with high cooling rates are required to prevent a decline in exercise performance. In the present study, cold water immersion of the hand and forearm during HT reduced and maintained a lower T_re_. It also improved intermittent exercise performance in a protocol that imitates intermittent exercise games. Cold water immersion of the hand and forearm is more convenient because it cools a small area compared with the whole body. Furthermore, considering that the actual and perceived heat stress could be removed in a short period of time (15 min), hand and forearm water immersion is a noteworthy practical cooling strategy in athletic settings.

## 5 Limitation

The first limitation of the present study is the reproducibility of intermittent athletic games. Ozgunen et al (2010) reported that in an actual soccer game, the peak of the T_c_ was observed at the end of the first half and remained lower than the peak value of the first half in the second half because the running distance in the second half was lower than that in the first half in the heat. However, the highest values of T_re_ in the present study were observed in the second half. These differed from the results obtained in the actual athletic field. In the present study, the exercise intensity of active recovery was similar in the first and second halves, which may have contributed to the increase in T_re_ in the second half. In addition, the actual exercise mode of intermittent team sports is running and not cycling. Therefore, it is necessary to investigate the actual exercise performance in the field in future research. Second, HT is mainly used for communication for tactical modifications and preparation for the second half (e.g., hanging clothes and re-warming up). In fact, the time that can be allocated to the cooling intervention is considered to be less than 15 min; therefore, it is also necessary to examine the effect of cooling in a shorter time. Finally, only male participants were included in this study. Considering that there are sex differences in body composition and thermoregulatory responses (Yanovich et al., 2020), the application of this method to females should be approached with caution.

## 6 Conclusion

It was concluded that cold water immersion of the hand and forearm during HT improved subsequent intermittent exercise performance. The cooling of the hand and forearm is thought to have caused a decrease in T_re_ and T_sk_, reduction in cardiovascular stress such as HR and SkBf, and decrease in subjective sensations, such as TS, TC, and RPE. These effects were observed within a short cooling time of 15 min. Since it is a convenient method, cold water immersion of the hand and forearm is noteworthy as a practical cooling strategy in athletic settings.

## Data Availability

The original contributions presented in the study are included in the article/Supplementary Material, further inquiries can be directed to the corresponding author.
