# Supplementary material for: Cold water immersion of the hand and forearm during half-time improves intermittent exercise performance in the heat
Source: Front Physiol. 2023 Jun 8;14:1143447. doi: 10.3389/fphys.2023.1143447 (PMC10285063; doi:10.3389/fphys.2023.1143447)
Supplement: Supplementary file 1 [file DataSheet1.PDF]

Supplementary table 1

		HT					Thrid					rest	Forth				
		3min	6min	9min	12min	15min	3min	6min	9min	12min	15min	2min	3min	6min	9min	12min	15min
T_{re}	p	0.607	0.206	0.023	0.003	0.000	0.000	0.000	0.000	0.000	0.000	0.000	0.000	0.000	0.000	0.000	0.000
	Cohen's d	-0.174	-0.470	-0.945	-1.393	-1.964	-1.870	-1.976	-1.943	-1.948	-2.007	-2.179	-1.977	-1.847	-1.684	-1.671	-1.594
T_{sk}	p	0.007	0.000	0.000	0.000	0.000	0.000	0.007	0.043	0.042	0.257	0.006	0.421	0.168	0.619	0.456	0.512
	Cohen's d	-1.516	-2.638	-2.892	-2.766	-2.396	-1.818	-1.172	-0.801	-0.627	-0.314	-0.676	-0.209	-0.379	-0.130	-0.192	-0.166
HR	p	0.001	0.000	0.000	0.000	0.001	0.000	0.001	0.002	0.012	0.004	0.008	0.142	0.111	0.135	0.876	0.187
	Cohen's d	-1.722	-2.238	-3.022	-3.517	-1.281	-1.538	-0.963	-0.709	-0.933	-0.883	-0.826	-0.708	-0.443	-0.416	-0.034	0.558
TS	p	0.007	0.005	0.005	0.005	0.005	0.003	0.004	0.010	0.006	0.004	0.013	0.006	0.026	0.026	0.041	0.041
	Cohen's d	-0.934	-1.919	-2.451	-2.607	-2.290	-2.311	-1.449	-1.150	-1.198	-1.355	-1.192	-1.441	-0.907	-0.984	-0.875	-0.938
TC	p	0.005	0.003	0.003	0.003	0.003	0.003	0.007	0.005	0.011	0.004	0.011	0.010	0.004	0.026	0.026	0.039
	Cohen's d	1.398	2.058	2.184	2.048	1.891	2.195	1.462	1.346	0.831	1.002	0.893	0.894	0.926	0.753	0.797	0.611
RPE	p	-	-	-	-	-	0.011	0.011	0.007	0.021	0.011	-	0.003	0.011	0.011	0.010	0.014
	Cohen's d	-	-	-	-	-	-1.480	-1.057	-1.179	-1.030	-1.024	-	-1.499	-0.955	-0.910	-0.882	-0.918
